# *Corynebacterium diphtheriae* Proteome Adaptation to Cell Culture Medium and Serum

**DOI:** 10.3390/proteomes9010014

**Published:** 2021-03-13

**Authors:** Jens Möller, Fatemeh Nosratabadi, Luca Musella, Jörg Hofmann, Andreas Burkovski

**Affiliations:** 1 Microbiology Division, Department of Biology, Friedrich-Alexander-Universität Erlangen-Nürnberg, 91058 Erlangen, Germany; fatemeh.nosratabadi@fau.de (F.N.); luca.musella@fau.de (L.M.); andreas.burkovski@fau.de (A.B.); 2 Biochemistry Division, Department of Biology, Friedrich-Alexander-Universität Erlangen-Nürnberg, 91058 Erlangen, Germany; joerg.hofmann@fau.de

**Keywords:** diphtheria, host-pathogen interaction, label-free quantification, metabolic pathway, proteomics

## Abstract

Host-pathogen interactions are often studied in vitro using primary or immortal cell lines. This set-up avoids ethical problems of animal testing and has the additional advantage of lower costs. However, the influence of cell culture media on bacterial growth and metabolism is not considered or investigated in most cases. To address this question growth and proteome adaptation of *Corynebacterium diphtheriae* strain ISS3319 were investigated in this study. Bacteria were cultured in standard growth medium, cell culture medium, and fetal calf serum. Mass spectrometric analyses and label-free protein quantification hint at an increased bacterial pathogenicity when grown in cell culture medium as well as an influence of the growth medium on the cell envelope.

## 1. Introduction

*Corynebacterium diphtheriae* is a gram-positive, non-motile, facultative anaerobe bacterium [1] which may lead to severe infections in humans. Symptoms of the classical upper respiratory tract diphtheria caused by diphtheria toxin (DT)-producing *C. diphtheriae* strains may range from mild pharyngitis with low fever to suffocation and death [2]. Due to effective vaccination strategies, diphtheria is generally well-controlled [3,4]. Nevertheless, 22,986 cases were reported by the World Health Organization with outbreaks in India (9622), Ethiopia (7184), Nigeria (2289), and Madagascar (1815) [4].

Besides infections by toxigenic strains, non-toxigenic strains can also cause severe symptoms in humans such as skin lesions [5], myocarditis, [6] and septic arthritis [7,8]. The latest data available showed an increase in infections caused by non-toxigenic strains at least in Germany [9]. Non-toxigenic strain ISS3319, used in this study, was isolated from a patient with severe pharyngitis/tonsillitis and was described as a strain with arthritogenic potential [10]. When mice were challenged with *C. diphtheriae* ISS3319, the bacteria were able to infiltrate deeper tissues, and joints of infected mice developed mild-to-moderate arthritis. Interestingly, the bacteria were also able to persist in kidneys and spleens for more than two weeks after infection [10].

To unravel mechanisms of host-pathogen interaction, a number of in vitro experiments with non-toxigenic corynebacterial strains and human and animal cell lines were carried out (e.g., see [10,11,12,13,14,15,16,17]). However, until now, the influence of cell culture medium and serum were completely neglected. To address this problem, we started a proteomics approach to characterize putative influences of different cell culture media on the proteome of *C. diphtheriae*.

Proteomics have already been used successfully in studying different pathogenic corynebacteria [18,19,20,21,22]. Nevertheless, most of the currently existing proteomic studies were carried out using bacteria grown in complex media; however, these do not represent the conditions the bacteria are facing during the infection process in vitro. For this purpose, the influence of two different cell culture media on viability, cell growth, and proteome of a non-toxigenic *C. diphtheriae* strain was analyzed. In total, 781 proteins were identified, 41 differentially expressed proteins and 115 proteins exclusively expressed in cell culture medium were found (49 proteins were exclusively observed for RPMI 1640, 35 proteins exclusive for FCS, and 31 proteins present in both conditions). The data suggest an increased bacterial pathogenicity when grown under stress conditions as well as an influence of the growth medium on the cell envelope. An increased occurrence of two-component systems indicates an adaption and stress response of the bacteria.

## 2. Materials and Methods

### 2.1. Bacteria and Growth Conditions

*C. diphtheriae* strain ISS3319 [10] (kindly provided by Christina von Hunolstein, Istituto Superiore di Sanità, Rome, Italy) was cultured under constant shaking in brain heart infusion (BHI) broth (Oxoid), RPMI 1640 (Capricorn, Epsdorfergrund, Germany), or fetal calf serum (FCS) (Capricorn, Epsdorfergrund, Germany) in bluffed flasks at 37 °C. For growth experiments an over-day culture was inoculated to an optical density at 600 nm (OD_600_) of 0.15 and grown at 37 °C under constant shaking. Colony forming units (CFU) and OD_600_ were determined every hour for 6 h and after 24 h. Experiments were carried out in three independent biological replicates, mean and standard deviation were calculated for every time point.

### 2.2. Sample Preparation of Proteomic Analysis

Protein samples were prepared as described [18]. Bacteria grown in BHI medium, RPMI 1640, and FCS were harvested for whole proteome analysis and lysed with a homogenizer using glass beads (5.5 m s^−1^, 30 s, 5 cycles, 4 °C). 40 µg of extracted proteins were alkylated (40 mM CAA final concentration), precipitated with acetone (80% final concentration, overnight, 4 °C), resuspended in 100 mM TEAB buffer with 2 µg trypsin, and digested overnight at 37 °C. Twenty-five micrograms of the resulting peptides (three biological replicates of secreted proteins and whole proteome from bacteria grown in BHI, RPMI 1640, and FCS) were purified using C18 stage tips, vacuum dried, and resuspended in 0.1% trifluoroacetic acid (TFA) before being supplied for LC-MS/MS analysis [18].

### 2.3. Mass Spectrometry

Mass spectrometric analysis was carried out as described previously [18,19,23]. For separation of peptides, 10 µg were supplied to a nanoflow Ultimate 3000 HPLC (Dionex, Sunnydale, CA, USA) and loaded onto an EASY-Spray column (Thermo Fisher Scientific; C18 with 2 µm particle size, 50 cm × 75 µm) with a flow rate of 200 nL min^−1^ and an increasing acetonitrile concentration over 120 min. An Orbitap Fusion mass spectrometer (Thermo Fisher Scientific, Bremen, Germany) was used for analysis of all samples using the following settings: spray voltage 2000 V, transfer tube temperature 275 °C, scan range for MS 1 detection in the Orbitrap 300–2000 (m/z), 50 ms maximum injection time, automatic gain control (AGC) target of 400,000, and Orbitrap resolution of 120.000. For collision-induced dissociation the most intense ions were selected with collision energy of 35%. For ion trap detection a maximum injection time of 250 ms and an AGC target of 100 were set [18,19,23]. The Proteome Discoverer program package 1.4 (Thermo Fisher Scientific, Bremen, Germany) and *C. diphtheriae* NCTC13129 database (Proteome Id: UP000002198) in UniProt (www.uniprot/proteomes) (accessed on 12 March 2021) were used to analyze the resulting raw data files with following settings: (I) theoretical masses of peptides were generated with a maximum of two missed cleavages, as described in [24], (II) fixed modification: carbamidomethyl on cysteine, (II) dynamic: oxidation of methionine, (III) mass tolerance for survey scans: 10 ppm and 0.6 Da for fragment mass measurements, (IV) false discovery rate (FDR): 1%. FDR was determined based on a target-decoy search and q-value the implemented percolator workflow from the Proteome Discoverer 1.4 software package (Thermo Fisher Scientific, Bremen, Germany) [19]. 

### 2.4. Data Analysis

Only proteins, which are present in all three independent biological replicates, were considered as identified. The peak areas of the identified proteins were normalized via the molecular weight. For label-free quantification, the relative abundance of each protein was calculated based on the total protein approach (TPA) method [25]. A multiple sample test (ANOVA) was applied to identify significant expression levels of the common proteins. A Z-score was calculated before hierarchical cluster analysis using the Euclidian algorithm. Information in respect to prediction location of identified proteins were extracted from published data [26]. Data on pathway analysis were extracted from Sangal et al., 2015 [26] and the KEGG consortium [27,28,29]. Collected data (see supplementary Table S1) were visualized using the proteomaps program (https://bionic-vis.biologie.uni-greifswald.de/) (accessed on 12 March 2021) [30,31,32].

## 3. Results

### 3.1. Growth Experiments under Different Culture Conditions

**Figure 1:**
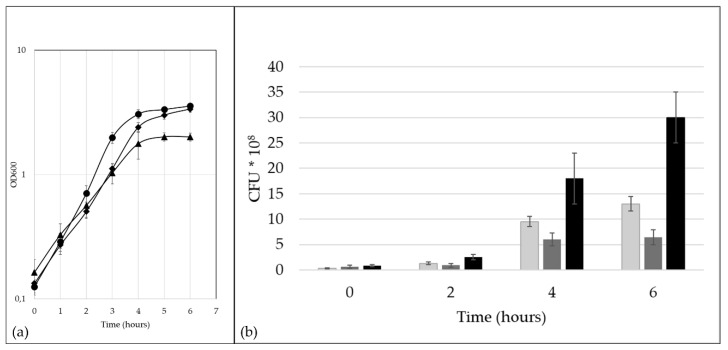
Growth of *C. diphtheriae* ISS3319. (**a**) Optical density in brain heart infusion (BHI) medium (rhombi), RPMI 1640 (triangles), and fetal calf serum (FCS) (circles). (**b**) Colony forming units (CFU) at different time points for bacteria grown in BHI medium (light grey), RPMI 1640 (dark grey), and FCS (black). Three independent biological replicates were carried out for all experiments. Data show the calculated mean value and the resulting standard deviation.

In order to evaluate the influence of standard cell culture media on bacterial growth, optical density and colony-forming units of ISS3319 cultures were monitored. Bacterial growth in standard BHI medium was comparable with growth in cell culture media, i.e., FCS and RPMI 1640 (Figure 1a). For bacteria grown in BHI a doubling time of 57 ± 2 min was determined, while 74 ± 3 min were measured for RPMI 1640 and 51 ± 2 min for FCS. After 24 h incubation bacteria reached a final OD_600_ of 5.3 ± 0.4 in BHI, 2.2 ± 0.2 in RMPI 1640, and 7.4 ± 1.4 in FCS. To monitor cell viability, bacteria were plated after two, four, and six h. The number of CFU increased from the beginning of the experiment (zero h) to four h under all conditions. No growth for bacteria in RPMI 1640 medium at six h was noticed, whereas bacteria in BHI and FCS were able to increase biomass (Figure 1b).

### 3.2. Mass Spectrometric Analysis

**Figure 2:**
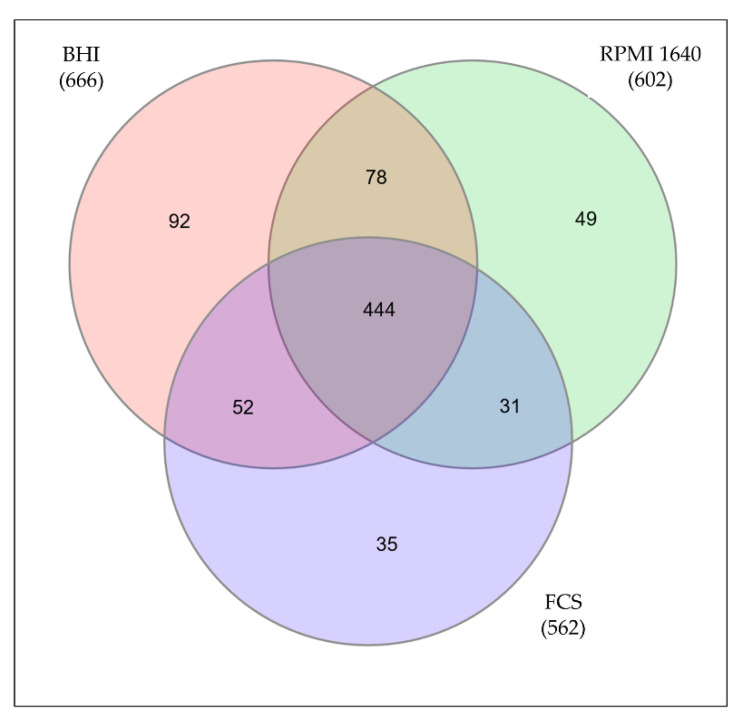
Proteins identified under different growth conditions. Only proteins identified in all three independent replicates were considered for further analysis. The Venn diagram shows the common proteins as well as the unique proteins for all three growth conditions.

By mass spectrometry, 666 proteins from *C. diphtheriae* ISS3319 grown in BHI medium were identified, 602 proteins from bacteria grown in RPMI 1640, and 562 proteins from bacteria grown in FCS. In total, 781 distinct proteins were found comprising 34.5% of the annotated genome. 92 proteins of the BHI sample, 49 proteins from RPMI 1640-grown cells, and 35 proteins from bacteria grown in FCS were found to be exclusive for the respective conditions (Figure 2, supplemental Table S2).

**Figure 3:**
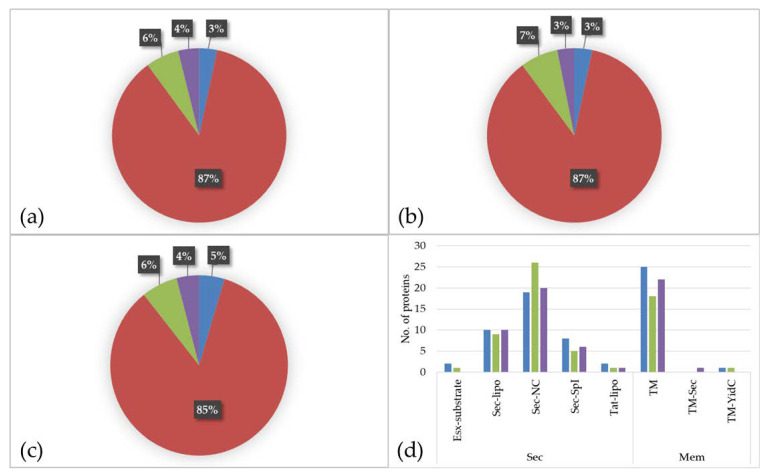
Proteome of *C. diphtheriae* strain ISS3319 grown in various culture media ((**a**): BHI; (**b**): RPMI 1640; (**c**): FCS). Percentage of different proteins located to the cytoplasm (red), extracellular (green), located to the membrane (purple), and proteins with ambiguous localization (blue). (**d**) shows the classification of secreted proteins and proteins with localization to the membrane regarding the secretion system. Blue: BHI, green: RPMI 1640 and purple: FCS.

The prediction of protein localization showed a similar distribution for all three media. The main parts of the identified proteins are located to the cytoplasm (BHI: 87%; RMPI 1640: 87%; FCS: 85%) followed by proteins with extracellular localization (BHI: 6%; RPMI 1640: 7%; FCS: 6%), proteins located to the membrane (BHI: 4%; RPMI 1640: 3%; FCS: 4%), and proteins with ambiguous localization (BHI: 3%; RPMI 1640: 3%; FCS: 5%) (Figure 3a–c).

An analysis of the secreted and transmembrane proteins revealed two ESAT-6-like proteins, EsxA (DIP0559) and EsxB (DIP0558), both present in BHI and the latter one also in RPMI 1640. These proteins belong to WGX100 family, which are type VII secretion substrates and thought to play a role in pathogenicity in taxonomically closely related bacteria such as *Mycobacterium tuberculosis* [26,33]. In total, 14 lipoproteins (Sec-lipo) (BHI: 10; RPMI 1640: 9; FCS: 10), 29 non-classical secreted (Sec-NC) proteins (BHI: 19; RPMI 1640: 26; FCS: 20), 10 proteins with an SPI signal peptide (Sec-SPI) (BHI: 8; RPMI 1640: 5; FCS: 6), and two lipoproteins secreted by Twin-Arginine Translocation pathway (TAT-lipo) were identified (BHI: 2; RPMI 1640: 1; FCS: 1). Within the Sec-SPI proteins are two putative secreted antigens and predicted mycolyltransferases involved in cell envelope assembly (DIP2193 and DIP2194) [34] and the putative invasion protein DIP1281, which was shown to be involved in virulence [35]. The TAT-lipo protein DIP1389 is a putative Dyp family peroxidase, which is thought to bind heme (Figure 3d).

When proteins with localization to the membrane (TM) were analyzed in total 32 proteins were found (BHI: 25; RPMI 1640: 18; FCS: 22). The multifunctional protein DIP0733, a fibronectin-binding protein involved in adhesion and invasion of host cells [17], was present in all three samples (BHI, RPMI 1640, and FCS). One single YidC protein (TM-YidC), namely DIP2379, which is involved in insertion of integral membrane proteins [36], and one transmembrane protein (DIP0736) with an SPI (TM-sec) was found in the dataset (Figure 3d).

### 3.3. Differentially Expressed Proteins in Cell Culture Media and Serum

**Figure 4:**
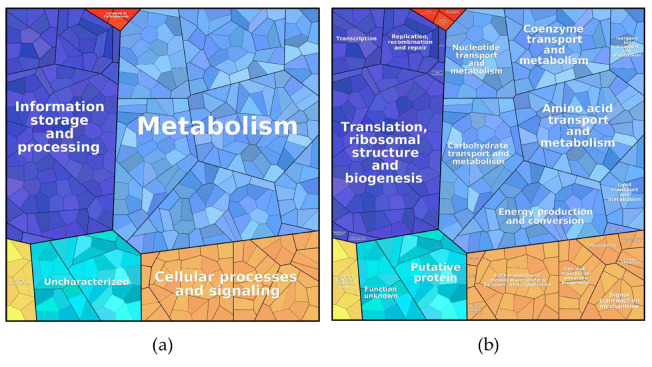
ProteoTreeMap of the total protein content in all three samples. (**a**) level 1: metabolic pathway and (**b**) level 3: function.

Proteins found under all three culture conditions were subjected to a pathway analysis (see ProteoTreeMap (Figure 4)). Most proteins were attributed to the categories metabolism, information storage and processing, and cellular processes and signaling as would be expected since proteins were extracted from similarly growing cells.

Interestingly, three proteins involved in pathogenesis were identified: the multifunctional protein DIP0733, the conserved hypothetical protein DIP1546, and the resuscitation-promoting factor RpfB DIP0874. Based on a label-free quantification method (TPA-method) the relative abundance of each proteins was calculated. The most abundant protein in all three samples was the iron repressible polypeptide (*dirA)* (DIP1420) and is involved in cellular processes and signaling. This protein has an ortholog protein AhpC in *M. tuberculosis* involved in tolerance of reactive oxygen and nitric oxide tolerance during macrophage infection [21,37,38]. The DtxR-regulated protein DIP2303 [39], which is involved in DNA protection during starvation, was among the most abundant proteins. This protein is similar to the starvation inducible non-specific DNA-binding protein (Dps) in *Escherichia coli* [40] and protects DNA from oxidative stress damage [41]. The manganese superoxide dismutase (*sodA)* DIP2261 also showed a high abundance in all three samples. SodA is involved in cell viability and knock-out mutants of *Corynebacterium melassecola* showed increased sensitivity to superoxide radicals [42].

**Figure 5:**
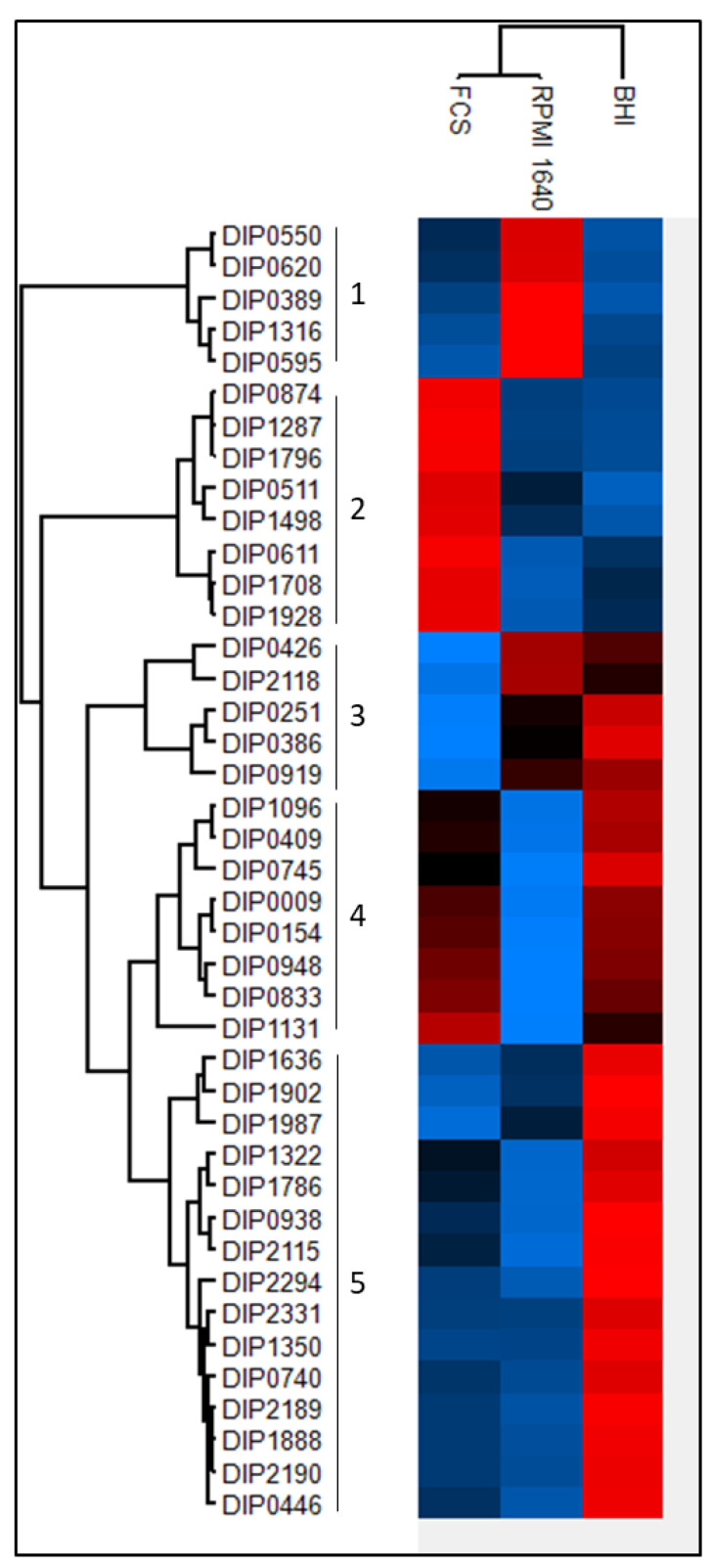
Differences in expression level of common proteins. A high intensity is shown in red and goes to a low intensity in blue. The relative abundance was used for a multiple sample test (ANOVA). A Z-score was calculated for the resulting 41 proteins and clustered using the Euclidian algorithm.

Based on the abundance of each protein a multiple sample test using the ANOVA algorithm was applied. Forty-one proteins showed significantly different expression patterns among the three different growth conditions. The hierarchical cluster analysis revealed a separation from bacteria grown in BHI from the bacteria grown in RPMI 1640 medium and FCS. The proteins can be divided into five main clusters (Figure 5; supplemental Table S3). In the first cluster are proteins with a high expression in RPMI 1640 and lower expression rates in BHI and FCS represented. Two proteins of this cluster, DIP0550 and DIP0620, are under the regulation of DtxR [39,42], which indicates iron starvation under these conditions.

The second cluster shows the enriched proteins when bacteria were grown in serum, and clusters 3, 4, and 5 show proteins with a lesser abundance in RPMI 1640 and FCS compared to BHI. The resuscitation-promoting factor RpfB (DIP0874) shows a high expression level in serum compared to BHI and RPMI 1640. Cluster four comprises eight proteins (DIP1096, DIP0409, DIP0745, DIP0009, DIP0154, DIP0948, DIP0833, and DIP1131). Significantly increased abundance of proteins from bacteria grown in BHI medium are represented in cluster five. This cluster includes 15 proteins (DIP1636, DIP1902, DIP1987, DIP1322, DIP1786, DIP0938, DIP2115, DIP2294, DIP2331, DIP1350, DIP0740, DIP2189, DIP1888, DIP2190, and DIP0446), three proteins (DIP0740, DIP2189, and DIP2190) are thought to be involved in mycolic acid synthesis [43] and a homologue of DIP1350 is essential in *M. tuberculosis* [44].

### 3.4. Newly Synthesised Proteins under Two Different Growth Conditions

**Figure 6:**
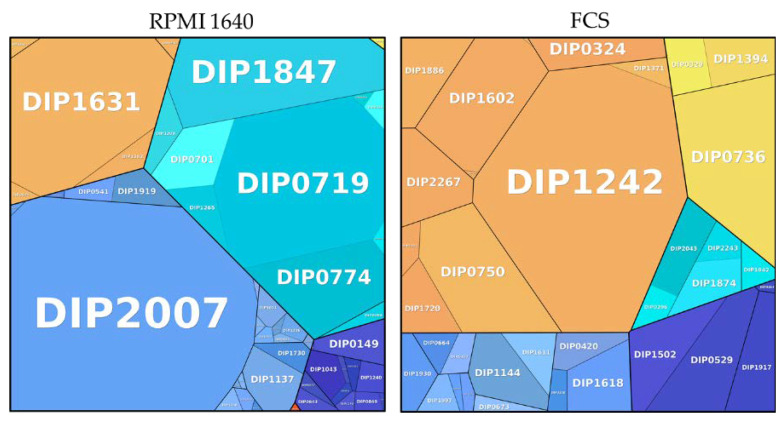
Metabolic pathways of proteins exclusively found in different growth conditions. Information storage and processing: dark blue; metabolism: light blue; cellular processes and signaling: orange; involved in pathogenesis: red; uncharacterized: turquoise; poorly characterized: yellow. The size of the area is proportional to the abundance calculated based on the total protein approach (TPA) method for label-free quantification.

The analysis of the proteins exclusively found in bacteria grown under different culture conditions (Figure 6; Table 1 and Table 2) revealed 92 proteins specific to BHI, 35 proteins for FCS, and 49 proteins for RPMI 1640.

Bacteria grown in RPMI 1640 showed an enrichment of proteins involved in metabolism (40.00%) followed by a similar amount of uncharacterized proteins (39.31%), proteins involved in cellular processes and signaling (15.94%), information storage and processing (4.65%), poorly characterized (0.08%), and one protein involved in pathogenesis (DIP1281).

Proteins involved in cellular processes and signaling were enriched (56.65%) in exclusive proteins from bacteria grown in FCS. Poorly characterized proteins were represented with a ratio of 14.07%, proteins involved in metabolism 12.90%, proteins involved in information storage and processing with 10.12%, and uncharacterized proteins with 6.24%.

A detailed breakdown of the function of the pathways from proteins exclusively found under growth conditions showed a high amount of ribosomal proteins (33.63%) in BHI, whereas an enrichment of proteins for inorganic ion transport and metabolism were noticed in RPMI (33.77%). One protein attributed to the category intracellular trafficking; secretion and vesicular transport contributed to almost one third (30.73%) of the exclusive proteins from FCS. DIP1242 is a Sec-independent protein translocase protein TatA, which is part of the twin-arginine (Tat) pathway. TatA was shown to be essential for protein export via the Tat pathway in *Corynebacterium glutamicum* [45].

To examine the influence of cell culture media on bacteria, high abundance proteins exclusively found in cells grown in RPMI 1640 and FCS were analyzed in more detail (Figure 6).

Among the 48 proteins found in RPMI 1640-grown bacteria, two were indicating iron limitation: The gene encoding the putative succinate dehydrogenase iron-sulfur protein DIP0372 (0.002%) was found in a predicted DtxR-regulated operon [39], and the gene of the uncharacterized protein DIP0579 (0.016%) also had has a DtxR binding site and was under the direct transcriptional control [46]. Five proteins were found to be involved in oxidative or nitrosative stress response (DIP0574: 0.003%; DIP1231: 0.004%; DIP1362: 0.044%; DIP1779: 0.002% and DIP1911: 0.011%). DIP0574 is identical to TsaD from mycobacteria and is upregulated under oxidative stress and contributes to pathogenicity of *M. tuberculosis* and *M. leprae* [47]. The thiol peroxidase Tpx (DIP1362) and the mycothiol acetyltransferase MshD (DIP1911) have homologues in *M. tuberculosis*. A study by Hu and Coates [48] showed that Tpx is involved in survival in macrophages and is crucial to establish acute and persistent infections with *M. tuberculosis*. Mutants of *mshD* have a limited stress tolerance and were not able to grow and survive in macrophages [49]. Homologous proteins of DIP0541 (0.002%), DIP0655 (0.006%), and DIP1634 (0.027%) play a role in infection and immune recognition in *M. tuberculosis* [50,51,52]. A Blast search of the sigma factor DIP0577 revealed an 83.6% identity with the RNA polymerase sigma-D factor from *Corynebacterium pseudotuberculosis*. SigD was found to affect the growth rate of *C. glutamicum* and regulates genes involved in mycolic acid synthesis [53].

The protein with the highest abundance in FCS is DIP1242 (30.07%), a Sec-independent protein translocase (*tatA*). A *tatA* mutant in *C. glutamicum* showed reduced cell growth and was susceptible to SDS [45]. Five proteins (DIP2043: 0.008%; DIP2005: 0.0001%; DIP1618: 2.74%; DIP1602: 6.82%; DIP0055: 6.8 × 10^−5^%) are involved in bacterial cell envelope formation. DIP1618 is a putative acyltransferase (PlsC) involved in lipid synthesis as shown for *C. glutamicum* [54]. Three proteins, the putative D-Ala D-Ala carboxypeptidase DIP2005, the putative secreted penicillin-binding protein PpbpA (DIP0055), and the UDP-N-acetylmuramoyl-tripeptide--D-alanyl-D-alanine ligase MurF (DIP1602) are involved in peptidoglycan synthesis and regulation of cell shape. A study by Arora and co-workers [55] showed that PpbpA functions in peptidoglycan biosynthesis in *M. tuberculosis* are involved in the regulation of bacterial cell length but do not affect growth. The putative secreted protein DIP2043 has a LytR_C domain, which is required for synthesis of anionic cell wall polymers [56] and present in bacterial cell wall assembly proteins. Five proteins are involved in membrane transport: the putative ABC transport system ATP-binding protein DIP1886 (2.70%), the protein-export membrane protein SecF (DIP1371, 0.002%), the putative uptake hydrogenase large subunit DIP0673 (0.002%), the Opt family protein (putative membrane protein) DIP0329 (0.005%), and the putative secreted protein DIP0324 (0.009%). Two proteins are involved in heme homeostasis (DIP2267 and DIP1394). The putative two component system response regulator protein HrrA (DIP2267: 3.23%) has a function for heme homeostasis [57,58] and the uncharacterized protein DIP1394 (0.009%) was identified as coproheme decarboxylase (ChdC) in *C. diphtheriae* [59]. Two probable drug targets were also exclusively present FCS, the probable nicotinate-nucleotide adenylyltransferase (NadD) DIP1775 and the SsrA-binding protein (SmpB) DIP0750. A study by Rodionova and co-workers [60] showed that NadD in mycobacteria acts as drug-target, and an in silico study by Jamal and co-workers identified DIP0750 as a putative drug target for corynebacteria [61]. A probable pathogenic function was found for two proteins in this data set: A putative serine-like trypsin protease (DIP0736: 10.51%) showed 30.86% identity to venom serine protease 2 (*vsp2*) from *C. ulcerans* 809. The putative protein DIP1144 (2.85%) shows 72.4% identity with PknD from *C. pseudotuberculosis*. PknD plays a role in pathogenesis of *M. tuberculosis* on the central nervous system. This tissue specific serine-threonine protein kinase triggers the invasion of brain endothelial cells [62].

When the two growth conditions, FCS and RPMI 1640, were compared to BHI, 115 proteins were found to be exclusively expressed (see supplemental Table S2). Of these 115 proteins, 49 proteins are exclusive for RPMI 1640, 35 proteins were exclusive for FCS, and 31 proteins were present under both growth conditions. An analysis of the latter revealed an involvement in bacterial stress response, pathogenicity, or increased antibiotic tolerance (Table 3). Interestingly, two proteins (DIP0600 and DIP2327) are two component response regulator proteins. The protein DIP0600 is part of an pathogenic island (PAI) with proteins involved in siderophore biosynthesis and transport, which includes the proteins DIP0597 and DIP0585, also found in both conditions [63]. Beside the putative iron transport system ATP-binding protein DIP0585, a second protein, the putative iron transport system exported solute-binding component protein DIP1086 was identified. Another protein which is part of a PAI, is the conserved hypothetical protein DIP0823 involved in cellular processes and signaling [64]. The homologue protein of DIP1225, RbpA in *M. tuberculosis*, was found to increase the resistance to the antibiotic rifampicin [65].

## 4. Discussion

In this study, we analyzed the proteome of the non-toxigenic *C. diphtheriae* strain ISS3319 grown under three different culture conditions. In total, we identified 781 proteins in all three data sets. 444 proteins were found under all three conditions, 92 were only found in BHI-, 49 proteins in RPMI 1640-, and 35 proteins in FCS-grown bacteria.

When the common proteins were analyzed in respect to their annotated function, the majority were involved in metabolism followed by information storage and processing. This may simply reflect the similar growth observed in standard complex medium (BHI), cell culture medium (RPMI 1640), and serum (FCS). In addition, specific proteome adaptation to cell culture medium and serum was observed.

Obviously, iron especially is a critical nutrient under the tested growth conditions. Bacteria grown in cell culture medium and serum expressed two proteins involved in iron acquisition, DIP1086 and DIP2327. DIP1086 is involved in iron acquisition and transport, and DIP2327 (ChrA) activates the gene expression of *hmuO* and *hrtAB* by the binding of the upstream regions of their promoters [66]. HmuO is a heme oxygenase and therefore degrades heme to acquire heme-iron sources [67]. Heme is the most abundant iron source of vertebrates to acquire iron, but it is also required as cofactor of many different enzymes, has a role in bacterial metabolism, and provides an advantage for bacteria during infection [68,69]. For this purpose, bacteria have to balance the intracellular heme concentrations. One protein from the novel identified coproporphyrin-dependent heme biosynthesis pathway, DIP1394, was identified in bacteria grown in FCS. It is speculated that heme biosynthesis occurs only in environments where iron is available [69,70,71]. It is probable that, due to the detection limit of the mass spectrometer, this protein was not detected in the data sets of bacteria grown in BHI or RPMI 1640. Despite the function of DIP1420 as putative virulence factor, the homologue protein AhpC was found to serve as heme-binding protein for storage [68,69]. DIP1420 was the most abundant protein under all examined growth conditions, suggesting an essential role in bacterial metabolism. Availability of heme, and therefore heme storage, might play an essential role in survival of bacteria in macrophages, since heme is a crucial component of catalases which protect bacteria against oxidative burst [69].

During bacterial infection of the host, macrophages are part of the first line defense against invading pathogens. A study by Weerasekera and co-workers [11] revealed high intracellular survival rates of *C. diphtheriae* strain ISS3319 in human and murine macrophages compared to other *C. diphtheriae* isolates. After bacteria are phagocytosed by macrophages, phagosomes are fused with lysosome to form phagolysosomes. Within the phagolysosome bacteria are killed by production of reactive oxygen species (ROS) [11]. Many bacteria evolved strategies to resist to this specific stress from macrophages [38,72,73]. For example, SodA protects *Salmonella* Typhimurium from killing by macrophages within the early infection stage [74]. Two proteins involved in survival under oxidative stress, SodA and Dps, were present in all three datasets, which hints at a high tolerance of the bacteria against oxidative stress under these growth conditions.

Virulence factors play a crucial role in bacterial pathogenicity. Three proteins involved in pathogenesis were present in all three data sets. The multifunctional protein DIP0733, the hypothetical protein DIP1546 and the resuscitation-promoting factor RpfB (DIP0874). DIP0733 is involved in adherence to erythrocytes [75] and human epithelial cells [76], in colonization and killing of *Caenorhabditis elegans*, binding to collagen and to fibrinogen. The ability of *C. diphtheriae* to bind to fibrinogen by DIP0733 is thought to play a role in evading the recognition by the host immune system [17]. The hypothetical protein DIP1546 was shown in a previous study from Ott and co-workers [77] to act as colonization factor in a *C. elegans* model system. Resuscitation-promoting factors (Rpf) are peptidoglycan-hydrolyzing enzymes and known to function in resuscitation and virulence of *M. tuberculosis* [78,79]. *C. diphtheriae* RpfB (DIP0874) showed significantly higher abundance in bacteria grown in FCS compared to bacteria grown in BHI or RPMI 1640 medium. Pathogenic islands (PAI) in pathogenic bacteria include disease- related factors which serves for antibiotic resistance, or proteins which are helpful for the current growth environment [80]. Twenty-four PAIs are reported in the genome of *C. diphtheriae* NCTC13129 [81]. Three proteins of a PAI, including one protein of a putative two component system response regulator (DIP0600) and two proteins involved in siderophore biosynthesis and transport (DIP0585 and DIP0597), were found in bacteria grown in RPMI 1640 and serum and not in bacteria grown in BHI.

A considerable number of known *C. diphtheriae* virulence factors was present under the three growth conditions tested, indicating a preadaptation of this species to host conditions. The putative invasion protein DIP1281 was exclusively found in RPMI 1640, while a Vsp2 homologous protein DIP0736 and a PknD homologous protein DIP1144, both involved in virulence of pathogenic corynebacteria [62,82], were found in the data set of bacteria grown in FCS. This suggests an influence of growth media on the virulence potential of *C. diphtheriae*.

## 5. Conclusions

In this study, the influence of cell culture media on bacterial growth and the bacterial proteome was investigated for *C. diphtheriae* strain ISS3319. The data suggest an increased virulence potential when bacteria are grown in cell culture medium and serum even without host cell contact. This result supports the idea that an influence of cell culture conditions on bacterial pathogenicity has to be considered in host-pathogen interactions studies.

## Figures and Tables

**Table 1:** Distribution of metabolic pathways of exclusive proteins found in the respective medium. The numbers show the abundance in percent [%].

Pathway	RPMI 1640	FCS
Cellular processes and signaling	15.94	56.65
Information storage and processing	4.65	10.12
Involved in pathogenesis	0.03	-
Metabolism	40.00	12.90
Poorly characterized	0.08	14.07
Uncharacterized	39.31	6.24

**Table 2:** Distribution of proteins exclusively found under two different growth conditions (RPMI 1640 and FCS). Pathway is shown in the first column (C: Cellular processes and signaling; I: Information storage and processing; P: Involved in Pathogenesis; M: Metabolism). The numbers represent the relative amount [%] when the exclusive proteins were set to 100%.

Metabolic Function	Pathway	RPMI 1640	FCS
Cell wall/membrane/envelope biogenesis	C	0.24	6.88
Cellular community-prokaryotes	C	-	2.41
Intracellular trafficking, secretion, and vesicular transport	C	-	30.73
Post-translational modification, protein turnover, and chaperones	C	15.50	10.71
Transporters	C	0.20	-
Signal transduction mechanisms	C	-	3.23
Defense mechanisms	C	-	2.70
Translation, ribosomal structure, and biogenesis	I	2.52	7.49
Replication, recombination, and repair	I	1.50	0.10
Transcription	I	0.62	-
Transfer RNA biogenesis	I	-	2.53
Cell wall/membrane/envelope biogenesis	P	0.24	6.88
Amino acid transport and metabolism	M	0.06	4.04
Coenzyme transport and metabolism	M	0.50	1.13
Nucleotide transport and metabolism	M	1.83	1.26
Inorganic ion transport and metabolism	M	33.77	1.34
Carbohydrate transport and metabolism	M	1.23	-
Lipid transport and metabolism	M	2.53	4.64

**Table 3:** Unique proteins found in bacteria grown in cell culture medium and serum. Data show the UniProt accession number, the gene onthology (GO), the abundance in RPMI and FCS in percent and a short description of the protein.

Accession No.	GO	RPMI [%]	FCS [%]	Description
Q6NJ28	DIP0585	0.001	0.011	Putative iron transport system ATP-binding protein
Q6NJ19	DIP0597	0.056	0.108	Uncharacterized protein
Q6NJ16	DIP0600	0.007	0.039	Putative two component system response regulator
Q6NIF4	DIP0823	0.081	0.205	Glutaredoxin domain-containing protein
Q6NHP5	DIP1086	0.004	0.006	Putative iron transport system exported solute-binding component
Q6NHB5	DIP1225	0.079	0.003	RNA polymerase-binding protein RbpA
Q6NEE8	DIP2327	0.019	0.006	Two-component response regulator ChrA

## Data Availability

Raw data files and .msf files of mass spectrometric analysis were deposited to the ProteomeXchange Consortium (http://proteomecentral.proteomexchange.org) (accessed on 12 March 2021) via the PRIDE partner repository [83]. Data are available via ProteomeXchange ID PXD024381. The excel output files are provided as a single supplementary file (supplementary material_Cd_ISS3319_proteomics_output_file). The proteotreemaps are accessible via following links: Figure 4: http://bionic-vis.biologie.uni-greifswald.de/result.php?jobID=16098528745332&version=UserSpec; Figure 6 (RPMI): http://bionic-vis.biologie.uni-greifswald.de/result.php?jobID=16093262330360&version=UserSpec; Figure 6 (FCS): http://bionic-vis.biologie.uni-greifswald.de/result.php?job_ID=16093270429281&-version=UserSpec.

## Supplementary Materials

The following are available online at https://www.mdpi.com/2227-7382/9/1/14/s1, in a single excel file entitled Supplementary material_Cd_ISS3319_proteomics. Table S1: ProteoTreeMap, Table S2: Identified proteins, Table S3: Analysis of differentially expressed proteins in all three data sets. The Supplementary material_Cd_ISS3319_proteomics_output_file includes the score, coverage, number of peptides, PSM and the area of each identified protein, of all three independent biological replicates (R1, R2 and R3) and under all three conditions (BHI, RPMI and FCS).

## Abbreviations

AGC	automatic gain control
BHI	brain heart infusion
CAA	chloroacetamide
CFU	colony forming units
DT	diphtheria toxin
DtxR	diphtheria toxin repressor
FCS	fetal calf serum
FDR	false discovery rate
GO	gene onthology
HPLC	High-performance liquid chromatography
LC-MS/MS	liquid chromatography mass spectrometry/mass spectrometry
OD	optical density
PAI	pathogenic island
ROS	reactive oxygen species
RPMI	Roswell Park Memorial Institute
TEAB	triethylammonium bicarbonate
TFA	trifluoroacetic acid
